# The association between blood pressure and whole blood methylmercury in a cross-sectional study among Inuit in Greenland

**DOI:** 10.1186/1476-069X-11-44

**Published:** 2012-07-02

**Authors:** Anni Brit Sternhagen Nielsen, Michael Davidsen, Peter Bjerregaard

**Affiliations:** 1 Centre for Health Research in Greenland, National Institute of Public Health, University of Southern Denmark, Copenhagen, Denmark; 2 The Section and Research Unit of General Practice, Institute of Public Health, University of Copenhagen, Copenhagen, Denmark; 3 Research Programme on Public Health in Denmark, National Institute of Public Health, University of Southern Denmark, Copenhagen, Denmark; 4 Department of Health, Greenland Government, Greenland, Denmark

**Keywords:** Whole blood mercury, Blood pressure, Hypertension, Inuit, Greenland

## Abstract

**Background:**

The Inuit in Greenland have a high average consumption of marine species and are highly exposed to methylmercury, which in other studies has been related to hypertension. Data on the relation between methylmercury and hypertension is limited, especially in populations subjected to a high exposure of methylmercury. We examined the relation between whole blood mercury and blood pressure (BP) in Inuit in Greenland.

**Methods:**

A cross-sectional population-based study among adult Inuit in Greenland was performed in 2005–2009. Information on socio-demography, lifestyle, BP, blood samples and clinical measurements was obtained – the latter after overnight fasting. BP was measured according to standardized guidelines. Whole blood mercury concentration was used as a marker of exposure. The analyses were restricted to Inuit aged 30–69 years with four Greenlandic grandparents (N = 1,861). Multivariate regression analyses with inclusion of confounders were done separately for men and women with the omission of participants receiving anti-hypertensive drugs, except for logistic regression analyses of the relation between mercury and presence of hypertension (yes/no).

**Results:**

The mean whole blood mercury level was 20.5 μg/L among men and 14.7 μg/L among women. In multivariate analyses adjusted for confounders, diastolic BP decreased with increasing mercury concentration. In men diastolic BP decreased significantly for each four-fold increase in mercury concentration (Beta = −0.04, standard error = 0.01, p = 0.001), while no relation between mercury and diastolic BP was found among women. For systolic BP, a similar non-statistically significant result was seen only for men (Beta = −0.02, standard error = 0.01, p = 0.06). A relation between mercury and hypertension was only found in men; the odds ratio for hypertension was 0.99 (95% CI: 0.98-0.99). No relation between quintiles of mercury and hypertension was found. The relationship between mercury and BP parameters may be non-linear: In analyses of quintiles of mercury the overall effect of mercury on BP parameters was only statistically significant for diastolic BP among men (Wald test, p = 0.01), however pairwise comparisons showed that some quintiles were not statistically different. This result is supported by LOESS modelling.

**Conclusions:**

No adverse associations between whole blood mercury and blood pressure were found. With increasing whole blood mercury concentrations, diastolic BP and the risk of hypertension decreased among men in the study: this may be explained by confounding by exercise or unknown factors.

## Background

Methylmercury bioaccumulates in meat and organs of fish and marine mammals. Marine food, especially seal, is the main source of mercury in humans in Greenland [1]; mercury in blood is therefore a marker of relatively recent consumption of marine meat (mercury has a blood half-life of about 70–80 days) [2]. Methylmercury exposure can adversely affect the central nervous system, especially neurobehavioural development in children. In some studies it has been shown to induce hypertension in animals and humans. Mercury is also a risk factor of cardiovascular diseases [3,4]. Conversely, a recent large, American case–control study with relatively low median mercury in toenails found no adverse cardiovascular effects of adults exposed to methylmercury from fish consumption [5]. A cross-sectional population-based study, performed in 1993–1995, of 259 adult Greenlandic Inuit with a geometric mean mercury concentration of 30.6 μg/L found no association between whole blood mercury and systolic blood pressure (SBP) or diastolic blood pressure (DBP) [1]. However, data on the association between mercury and hypertension is limited, as concluded by Virtanen et al. in a review on mercury as a risk factor of cardiovascular diseases, which included studies from 1995–2005 [2].

It has been suggested that the relationship between the level of mercury and blood pressure (BP) is non-linear: higher levels of mercury are not always associated with higher values of BP. In a follow-up study among seven-year-old Faroese children exposed to methylmercury in utero, Sorensen et al. found that SBP and DBP increased linearly with cord blood mercury concentrations of up to 10 μg/L [6]. Above this level, no further increase in BP was observed. The same cohort was examined at age 14: no relation between cord blood mercury concentrations and BP was found [7].

Studies on adults do not agree on the association between mercury and BP. In another cross-sectional study among Greenlandic Inuit, increasing levels of blood mercury were associated with decreasing DBP and increasing pulse pressure (PP), but not SBP [8]. A population-based study among women from the US with relatively low blood mercury concentrations (mean 1.8 μg/L) reached a similar conclusion. At the highest quintile of mercury (2.1-21.4 μg/L) SBP was significantly lower among fish consumers than among non-fish consumers; a similar pattern, statistically non-significant, was seen for DBP [9]. A cross-sectional study among inhabitants of the Brazilian Amazon with a mean hair mercury concentration of 17.8 μg/g found the opposite: higher mercury levels were associated with increasing SBP, but not with DBP [10]. A follow-up study of Finnish men found that those in the upper tertile of mercury in hair (>2.03 μg/g) had a higher risk of coronary heart disease, cardiovascular disease, acute coronary events, and all-cause mortality than those in the two lower tertiles. The authors concluded that the protective role of n-3 fatty acids on cardiovascular health seems to be negated when mercury concentrations are high [4]. A cross-sectional study by Valera et al. [11] of 732 Inuit from Nunavik, Canada, with a mean blood mercury concentration of 10.4 μg/L also found that blood mercury was positively associated with SBP and PP; however, in contrast to the studies by Pedersen et al. [8] and Fillion et al. [10] no relation was found between mercury and DBP. In their analyses Valera et al. adjusted for the potential effect of some of the other nutrients in fish and seafood. A recent study by Choi et al. [12] recommends the inclusion of selenium and n-3 fatty acids in the analysis of the relation between mercury and BP, since these factors may affect BP (although in the opposite direction).

The lack of agreement between studies on the relationship between methylmercury and hypertension may depend upon:

a) the participants’ mercury level [12], i.e. there is a lack of studies of higher concentrations of mercury, which could possibly add new perspectives; and

b) the confounders included in the analysis. Some studies adjusted for the protective effect that eating fish may have on BP (e.g., selenium and PUFA may have a protective effect on cardiovascular disease by affecting BP in the opposite direction to the effect of mercury on BP [12]).

The purpose of the present paper is to examine the relation between blood mercury and BP in adult Inuit in Greenland, whose average consumption of marine species is significantly higher than that of adults in Western countries, allowing for the influence of markers of n-3 fatty acids and other potential confounders.

## Methods

### Participants

The total population of Greenland is 57,000 (2007), of whom 90% are ethnic Greenlanders (Inuit). Genetically, Greenlanders are Inuit (Eskimos) with an admixture of European genes; and closely related to the Inuit and Yupik in Canada, Alaska and Siberia.

Data stems from a cross-sectional population-based study among Inuit in Greenland performed from 2005 to 2009 in 18 towns and villages. A two-step sampling procedure was applied. First, the 17 towns and 60 villages in the country were stratified into larger towns (populations ≥ 2000), smaller towns and villages, and grouped by geographical location (south, mid, north and east Greenland). In each of the 10 resulting strata, 1–2 towns and 2–3 villages were randomly selected. In towns, a random population sample of 11-22% was drawn from the population registers in order to obtain approximately 300 participants; in villages all persons ≥ 18 years were eligible for participation. A full description of the population sample and survey methods is available [13]. The final sample is regarded as representative for Greenland, according to community size and geographical location, even though women more often participated than men and young men in particular were underrepresented. The participation rate was 67.5% for Greenlanders (in the clinical part of the study, 2005—2009).

Because genetic Inuit heritage might influence the relationship between mercury and BP, and BP may be higher among elderly participants, we only included persons between 30 and 69 years with four Greenlandic grandparents.

Ethical approval was obtained from the Ethical Review Committee for Greenland. Informed consent was obtained in writing from each participant.

### Measurements

Information on age, sex, smoking behaviour, the number of parents and grandparents that were Greenlanders or other nationalities (a marker of genetic Inuit heritage), and the drug-therapy (medications being taken) were recorded in an interviewer-administered questionnaire. The questionnaire was developed in Danish, translated into Greenlandic, back translated and revised.

Blood samples were obtained after overnight fasting. The whole blood samples for analysis of mercury and selenium were frozen at −20 °C and were analysed at Laboratoire de Toxicologie/INSPQ, Québec by inductively coupled mass spectrometry; the detection limit was 0.5 nmol/l, and 0.1 μmol/l, respectively. Before analysis blood samples were diluted 20-fold in a solution containing ammonium hydroxide. The fatty acid composition of the erythrocyte membranes was measured after membrane purification, chloroform/methanyl lipid extraction and methylation of fatty acids, followed by capillary GLC using a DB-23 column in a Hewlett-Packard GC chromatograph (which was carried out at Université Laval, Quebec).

BP was measured three times with the participant in a sitting position, after at least five minutes of initial rest and having not smoked for at least 30 minutes, using an automatic BP apparatus (Kivex UA 779) with cuffs matched to fit the participants’ arms. The two last measurements were averaged for the analyses. Waist circumference (WC) was measured on the standing participant midway between the iliac crest and the costal margin to the nearest millimetre.

### Definitions

Smoking behaviour was categorised into current smokers and non-smokers.

Pulse pressure (PP) was based upon systolic blood pressure (SBP) minus diastolic blood pressure (DBP).

Hypertension was defined as BP ≥ 140/90 mmHg or usage of anti-hypertensive drugs according to guidelines for the prevention of ischemic heart disease in general practice [14]: diuretics (ATC-code C03), beta-blockers (ATC-code C07), calcium channel blockers (ATC-code C08), angiotensin-converting enzyme (ACE-)inhibitors (ATC-code C09A), or angiotensin II antagonist (ATC-code C09C). If the participant used any of the medication(s) mentioned above it was taken as a surrogate for having ‘hypertension’ even if their BP was not ≥ 140/90 mmHg.

Age, WC, smoking, blood selenium and the ratio of n-3/n-6 fatty acids in red blood cell membranes were included as possible confounders, since in our data and/or published studies these were associated with both blood mercury and blood pressure [10-12,15]. There is controversy about whether n-3/n-6 ratio or n-3 concentration is the better risk factor in cardiovascular disease [16]. In the absence of a specific hypothesis for the possible blood pressure lowering effect of marine fatty acids we wished to control for intake of marine food and not specifically for any single component of the marine food. In our study the association between reported intakes of marine fats was more closely associated with n-3/n-6 ratio than with the absolute percentage of n-3 fatty acids and accordingly we chose the former for confounder control. We excluded information on total cholesterol, triglycerides, insulin resistance, and fasting plasma glucose as possible confounders, since it is not biologically plausible that they could confound the mercury-BP relationship.

### Statistical analysis

All analyses were performed for men and women separately. Initially all involved variables are presented stratified by sex. Arithmetic means with 95% confidence interval (CI) are given for continuous normally-distributed variables (tested by q-q plot) and as natural log-transformed geometric means for non-normally distributed variables. Since some studies provide median whole blood mercury this is also provided. Categorical data are presented as proportions. Differences between men and women were assessed using a *t*-test for continuous variables and with a chi-squared test for categorical variables.

SBP, DBP, and PP were analysed in an ordinary linear regression model using the log-transform of these variables. When analysing the relation between mercury and hypertension we used a logistic regression model. In the analyses of the relation between mercury and BP we omitted the participants who used anti-hypertensive drugs, since these might mask a possible relation between the exposure (mercury) and the outcome (BP). However, these participants were included in the analysis of the relation between mercury and hypertension (see “definitions”). We included mercury concentration in the regression analyses in two ways: either as a continuous base 4 log-transformed variable or grouped in quintiles (using a base 4 log-transformation means that we get a four-fold effect of mercury on BP, for example the effect on DBP if the concentration of blood mercury increases from 1 μg/L to 4 μg/L or from 8 μg/L to 32 μg/L).

First we studied the relationships between mercury and BP parameters using the Pearson coefficient of correlation, or Spearman’s test if data were not normally distributed, and performed simple regression analyses for every possible confounding variable that we had in mind when choosing variables to be entered into the models (see ‘definitions’) to test whether the variable was related to both the exposure and outcome. Variables systematically associated with BP *and* mercury (p ≤ 0.25) were included in the analyses. We included smoking habits in all analyses, since smokers have a relatively lower BP than usual, owing to the smoking restriction (see ‘measurements’). We did no model reduction in the multivariate analyses. A Wald test was used to assess the overall significance of quintiles of mercury with the lower quintile as reference.

Models were checked using Q-plots to assess the normality of residuals (with a mean of zero) from the linear models, and analyses of collinearity between variables were performed to avoid the inclusion of two variables highly correlated (Variance Inflation Factor values below 10 indicates no collinearity between variables). We checked the discriminative power of logistic regression models using the c-statistic, which is equivalent to the area under a receiver operating characteristic curve. All analyses were performed with the software PROC GLM and PROC LOGISTIC; (SAS, version 9.1, SAS Institute Inc).

## Results

A total of 1,861 participants with Greenlandic grandparents were included in the analyses. Baseline data are presented in Table 1, and showed an overrepresentation of female participants, who are younger, and with significantly lower levels of mercury, BP and PP than men. Median whole blood mercury among men was 22 μg/L (interquartile range (IQR): 11.0-41.0); among women 16 μg/L (IQR: 7.8-30.1), and for both men and women it was 18 μg/L (IQR: 8.8-34.1). Fewer women than men were current smokers (64.2% vs. 70.3%, p < 0.0006).

**Table 1:** **Characteristics of Inuit participants (30–69 years) with four Greenlandic grandparents**. Values are arithmetic means with a 95% CI for the mean unless stated otherwise

	**Men**			**Women**			P-value*	**Total**
**Characteristics**	N	Mean (95% CI)	Total range	N	Mean (95% CI)	Total range		Mean (95% CI)
Age (years)	812	48.8 (48.1-49.5)	30.0-69.9	1049	46.9 (46.4-47.5)	30.0-69.9	<0.0001	47.8 (47.3-48.2)
Systolic blood pressure (mmHg) ^§^	795	133.5 (132.2-134.8)	86.5-234.0	1025	124.3 (123.2-125.4)	74.0-221.0	<0.0001	128.3 (127.4-129.1)
Diastolic systolic blood pressure (mmHg) ^§^	795	81.7 (80.8-82.5)	43.5-140.0	1025	77.3 (76.6-78.0)	41.5-121.0	<0.0001	79.2 (78.6-79.7)
Pulse pressure (mmHg) ^§^	795	50.6 (49.7-51.5)	20.0-134.0	1025	45.8 (45.0-46.5)	20.0-125.0	<0.0001	47.8 (47.2-48.4)
Hypertension^a^, %	796	41.7		1029	31.3		<0.0001	35.8
Whole blood Mercury (μg/L) ^§^	805	20.5 (19.1-22.0)	0.4-280.0	1040	14.7 (13.8-15.6)	0.05-170.0	<0.0001	17.0 (16.2-17.8)
Quintiles of blood mercury (μg/L)^§^							^b^	
Quintile 1	161	4.78 (4.37-5.23)	0.4-8.7	209	3.33 (3.00-3.70)	0.05-6.4		
Quintile 2	173	12.75 (12.38-13.13)	8.8-17.0	213	9.20 (8.97-9.43)	6.6-12.0		
Quintile 3	151	21.65 (21.21-22.11)	17.3-26.1	204	15.81 (15.48-16.14)	12.2-20.1		
Quintile 4	159	35.43 (34.46-36.44)	27.0-49.0	206	26.54 (26.00-27.09)	21.0-35.0		
Quintile 5	161	81.07 (76.31-86.13)	50.0-280.0	208	54.63 (52.01-57.38)	36.0-170.0		
Total n-3 fatty acids	690	10.61 (10.27-10.94)	1.16-22.39	917	10.5 (10.23-10.76)	0.73-20.3	0.61	10.54 (10.34-10.75)
Total n-6 fatty acids	690	19.23 (18.88-19.59)	6.97-31.36	917	20.04 (19.74-20.33)	8.79-32.53	0.0006	19.69 (19.46-19.92)
Ratio (n-3)/(n-6) ^§^	690	0.51 (0.49-0.53)	0.07-2.11	917	0.49 (0.47-0.50)	0.07-1.59	0.12	0.50 (0.48-0.51)
Blood selenium (μg/L)^§^	801	292.41 (278.38-307.16)	77.0-5600.0	1037	279.60 (268.97-290.65)	68.0-4600.0	0.16	285.11 (276.52-293.98)
Waist circumference (cm) ^§^	795	92.7 (91.8-93.5)	64.0-160.0	1033	91.3 (90.5-92.2)	62.5-137.8	0.04	91.9 (91.3-92.5)

^*^Statistics: Comparison between men and women were performed using a *t*-test for continuous variables and Chi-square tests for categorical variables.

^§^ For non-normally distributed variables the geometric means are presented.

^a^ Hypertension was defined as SBP ≥ 140 mmHg or DBP ≥ 90 mmHg or the use of anti-hypertensive drugs.

^b^ Quintiles of whole blood mercury have been created separately for men and women. Therefore no comparisons have been made.

Blood mercury was not normally distributed, and Spearman’s test was used for correlation analyses among participants not receiving anti-hypertensive drugs. Blood mercury was positively correlated with age (men: ρ = 0.25, p < 0.0001; women: ρ = 0.28, p < 0.0001), selenium (men: ρ = 0.58, p < 0.0001; women: ρ = 0.59, p < 0.0001), and the ratio of n-3/n-6 fatty acids (men: ρ = 0.68, p < 0.0001; women: ρ = 0.69, p < 0.0001). Among men no correlation between blood mercury and SBP (ρ = −0.03, p = 0.43) and PP (ρ = 0.06, p = 0.11) was found; but mercury was negatively correlated to DBP (ρ = −0.13, p = 0.0004) and to WC (ρ = −0.14, p = 0.0001). Among women mercury was positively correlated to SBP (ρ = 0.09, p = 0.007) and PP (ρ = 0.13, p < 0.0001), but not to DBP (ρ = −0.02, p = 0.52) and WC (ρ = −0.03, p = 0.34). All of the variables chosen as possible confounders (age, blood selenium, smoking habits, ratio of n-3/n-6 fatty acids, and WC) were included in the multivariate analyses. None of these variables were associated with BP and mercury (p > 0.25) among both men and women.

Table 2 shows the results of linear regression analyses between blood mercury and BP parameters among participants not receiving anti-hypertensive drugs, allowing for the influence of age and other confounders. Among both sexes no statistically significant relation was found for mercury and PP and SBP. A negative relation between mercury and DBP was found among men; DBP decreased with each four-fold increase in mercury concentration (Beta = −0.04, standard error (S.E.) = 0.01). Among women no statistically significant relation was found for mercury and DBP (Beta = −0.01, S.E. = 0.01).

**Table 2:** **The association between whole blood mercury and blood pressure parameters among Inuit**^**§**^**(30–69 years) in Greenland; the analyses have been adjusted for confounders**^**ab**^

	**Log-Systolic blood pressure**^**c**^		**Log-Diastolic blood pressure**^**c**^		**Log-Pulse pressure**^**c**^	
Log whole blood mercury*4	Parameter estimate (standard error)	P-value	Parameter estimate (standard error)	P-value	Parameter estimate (standard error)	P-value
Men	−0.02 (0.01)	0.06	−0.04 (0.01)	0.001	0.01 (0.02)	0.57
Women	−0.003 (0.01)	0.77	−0.01 (0.01)	0.19	0.01 (0.02)	0.49

^§^ Only participants with four Greenlandic grandparents and with no anti-hypertensive drug therapy.

^a^ Confounders: age, smoking habits, blood selenium, ratio of n-3/n-6 fatty acids, waist circumference.

^b^ The models were based on 615 men and 787 women (with non-missing values for all included variables).

^c^ The outcome variables of interest, systolic and diastolic BP, and pulse pressure were not normally distributed and were included as natural log-transformed variables in the model.

The relationship between quintiles of mercury and BP parameters adjusted for age and other confounders are presented in Table 3. The overall effect of mercury (Wald-test) was statistically significant for DBP among men and for PP among women. Among women a tendency of decreasing PP with increasing mercury quintiles was found when comparing quintiles 2 and 3 with the lowest quintile (Wald test, p = 0.01), although only quintile 3 was statistically significantly lower compared to the lowest quintile. The relation between mercury and DBP among men decreased from quintile 3 to quintile 5 as compared to the lowest quintile, although the pairwise tests showed that only those in the two upper quintiles had a statistically significant lower DBP as compared to the lowest quintile. Among women the DBP was not significantly higher in mercury quintiles 2–5 vs. quintile 1. Analyses of residuals of the final linear models (Tables 2 and 3) showed that the linear regression assumption was fulfilled. In all final models we found no indication of collinearity between variables (all VIF values were <3).

**Table 3:** **The association between quintiles of whole blood mercury and blood pressure parameters among Inuit**^**§**^**(30–69 years) in Greenland, the analyses are adjusted for confounders**^**ab**^

	Log-systolic blood pressure*		Log-diastolic blood pressure*		Log-pulse pressure*	
**Whole blood mercury (μg/L)**	(exp)Parameter estimate (95% CI)	P^c^ (P^d^)	(exp)Parameter estimate (95% CI)	P^c^ (P^d^)	(exp)Parameter estimate (95% CI)	P^c^ (P^d^)
***Men***						
Quintile 1 (range: 0.4-8.7)	1	(0.45)	1	(0.01)	1	(0.51)
Quintile 2 (range: 8.8-17.0)	1.00 (0.97-1.03)	0.87	1.00 (0.96-1.03)	0.86	0.99 (0.94-1.05)	0.80
Quintile 3 (range: 17.3-26.1)	0.98 (0.94-1.01)	0.17	0.97 (0.93-1.01)	0.12	0.98 (0.92-1.04)	0.50
Quintile 4 (range: 27.0-49.0)	0.98 (0.94-1.01)	0.19	0.94 (0.90-0.98)	0.007	1.02 (0.96-1.09)	0.52
Quintile 5 (range: 50.0-280.0)	0.97 (0.93-1.01)	0.15	0.93 (0.89-0.98)	0.004	1.03 (0.96-1.11)	0.38
***Women***						
Quintile 1 (range: 0.05-6.4)	1	(0.17)	1	(0.19)	1	(0.01)
Quintile 2 (range: 6.6-12.0)	0.99 (0.97-1.02)	0.69	1.01 (0.98-1.04)	0.70	0.97 (0.93-1.03)	0.33
Quintile 3 (range: 12.2-20.1)	0.98 (0.96-1.01)	0.26	1.01 (0.97-1.04)	0.71	0.94 (0.89-0.997)	0.04
Quintile 4 (range: 21.0-35.0)	1.02 (0.99-1.05)	0.30	1.00 (0.97-1.04)	0.94	1.03 (0.97-1.10)	0.32
Quintile 5 (range: 36.0-170.0)	0.99 (0.95-1.02)	0.52	0.97 (0.93-1.01)	0.11	1.02 (0.95-1.10)	0.55

^§^ Only participants with four Greenlandic grandparents and with no anti-hypertensive drug therapy.

^a^ Confounders: age, smoking habits, blood selenium, ratio of n-3/n-6 fatty acids, waist circumference.

^b^ The models were based on 615 men and 787 women (with non-missing values for all included variables).

^c^ The outcome variables of interest, systolic and diastolic BP, and pulse pressure, were not normally distributed and were included as natural log-transformed variables in the model. The parameter estimate is presented as the antilog of the result of the linear regression analyses.

^d^ P-value of a “pairwise” comparison with the first quintile of whole blood mercury.

^e^ P-value of a Wald-test for the overall effect of mercury on blood pressure and pulse pressure.

**Figure 1:**
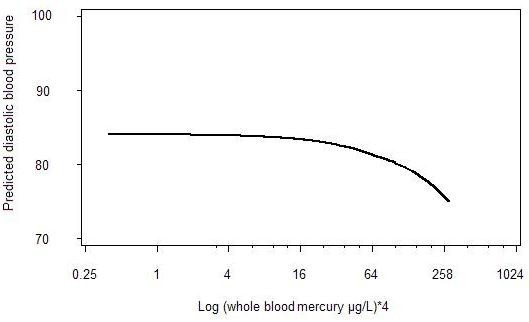
Relation between whole blood mercury (log base 4) and diastolic blood pressure among Inuit men (30–69 years) in Greenland*. * The shape was smoothed using locally weighted regression (LOESS) and restricted to men with blood mercury ≤ 300 μg/L, with four Greenlandic grandparents and with no anti-hypertensive drug therapy.

Since the results indicate that the relation between mercury and DBP is non-linear we examined the shape of that relation using locally weighted regression smoother (LOESS). Figure 1 shows that the DBP among men is almost stable until blood mercury concentrations are around 16 μg/L, following which there is a steady decrease. A similar pattern was seen for women (results not shown).

The logistic regression of the association between blood mercury as a continuous variable and hypertension (yes/no) indicated a negative relation among men (results not shown in a table); the odds ratio (OR) for having hypertension adjusted for confounders was 0.99 (95% CI: 0.98-0.99, p = 0.03). Among women no relation between mercury and hypertension was found (OR: 1.00 (95% CI: 0.99-1.01; p = 0.65).

The effect of mercury grouped in quintiles on hypertension is presented in Table 4. Among men the overall effect of mercury on hypertension (Wald test) was not statistically significant with the inclusion of confounders (p = 0.22), and the odds-ratio for hypertension did not increase linearly as mercury increased. Similarly, for women the relationship between mercury and hypertension was statistically insignificant in all the analyses. The results of the sensitivity analyses of the final logistic model showed that the c-statistics were between 0.74-0.77, which indicates a fair power of discrimination since the values are between 0.5 and 1.0.

**Table 4:** **Odds for hypertension among Inuit**^**§**^**(30–69 years) in Greenland vs. quintiles of whole blood mercury. The analyses are adjusted for confounders**^**ab**^

	**Hypertension (1 = hypertension, 0 = no hypertension)**^c^	
**Whole blood mercury (μg/L)**	Odds ratio (95% CI)	P^d^ (P^e^)
***Men***		
Quintile 1 (range: 0.4-8.7)	1	(0.22)
Quintile 2 (range: 8.8-17.0)	1.04 (0.62-1.73)	0.88
Quintile 3 (range: 17.3-26.1)	0.65 (0.37-1.15)	0.14
Quintile 4 (range: 27.0-49.0)	0.84 (0.45-1.57)	0.59
Quintile 5 (range: 50.0-280.0)	0.53 (0.26-1.10)	0.09
***Women***		
Quintile 1 (range: 0.05-6.4)	1	(0.60)
Quintile 2 (range: 6.6-12.0)	1.29 (0.77-2.18)	0.34
Quintile 3 (range: 12.2-20.1)	1.09 (0.63-1.89)	0,75
Quintile 4 (range: 21.0-35.0)	1.51 (0.85-2.69)	0.16
Quintile 5 (range: 36.0-170.0)	1.39 (0.72-2.70)	0.33

^§^ Only participants with four Greenlandic grandparents.

^a^ Confounders: age, smoking habits, blood selenium, ratio of n-3/n-6 fatty acids, waist circumference.

^b^ The models were based on 663 men and 889 women (with non-missing values for all included variables).

^c^ Hypertension was defined as BP ≥ 140/90 mmHg or usage of anti-hypertensive drugs.

^d^ P-value of a “pairwise” comparison with the first quintile of whole blood mercury.

^e^ P-value of a Wald test for the overall effect of whole blood mercury on hypertension.

## Discussion

The main finding of this cross-sectional study of the relationship between blood mercury and BP among 1,861 adult Inuit men and women (30–69 years) in Greenland was that increasing blood mercury concentrations were associated with decreasing DBP among men. Among women no statistically significant relation was found between mercury concentration and BP parameters. Analyses including quintiles of mercury suggest that the relationship between mercury and BP parameters may be non-linear throughout the concentration range. All the results were controlled for possible confounders. An additional smoothed regression curve of the relation indicates almost no changes in DBP for fairly low mercury concentrations, followed by a steady decrease.

### Study strengths and limitations

The strengths of this study are the large sample of adult Inuit men and women. This is the largest national dataset to report blood mercury measurements among adult Inuit. We measured BP with an automatic BP apparatus, which may be a more precise measurement than a sphygmomanometer; especially when several persons are involved in collection of data. We omitted participants receiving anti-hypertensive drugs in the analysis of mercury and BP; inclusion of these participants could have masked a relation between the exposure and outcome. We included information on n-3 fatty acids (as the ratio of n-3/n-6 fatty acids) in the statistical models as confounder, which may have an effect on BP in the opposite direction than mercury, and blood selenium that is also an anti-oxidative substance reflecting the intake of traditional Greenlandic food.

Some limitations must be considered. We measured BP three times, but some participants’ BP may be unreliable, owing to the white coat effect,^a^ and a 24-hour BP measurement is recommended to obtain more reliable test results. However, owing to the survey set-up this was impossible. The participants rested at least five minutes before the first BP measurement, and we used the average of the second and third BP measurements. We did not include information on physical activity, which could have decreased our participants’ BP; and hunters in Greenland, who may be among those in the highest quintile of mercury, in general have a high level of physical activity. However, we did not include physical activity as a confounder in our analyses, since information on physical activity is not yet available in our study. This is a cross-sectional study and therefore any interpretation about exposure and outcome should be carefully interpreted.

### Relation to other studies

Like Pedersen et al. [8], who studied mercury in blood and 24-hour BP among Greenlanders and Danes, we also found that DBP decreased with increasing blood mercury concentrations; however, this was only among men. This overall effect of mercury was also only present among men for quintiles of mercury (Wald test, p = 0.01). Among women we found no statistically significant lower DBP among those in the highest quintile compared with those in the lowest quintile.

Pedersen et al. [8] divided blood mercury into quintiles; the lower quintile included Danes, and the other quintiles included Inuit Greenlanders (with a median blood mercury = 16.2 μg/L). However they did not stratify these analyses by sex, as we did, which may explain why we found a difference in DBP among men for quintiles of mercury but not among women, since men in our study had a higher median blood mercury concentration (22 μg/L) than the median in their study, whereas the median blood mercury concentration among women in our study was almost similar (16 μg/L). However, Pedersen et al. found no difference in BP among the three mercury quintile groups including Greenlanders, and there was no relation between blood mercury and DBP when the Danes and Greenlanders were analysed separately (multiple adjusted linear regression analysis). Our results partially agree on this; we found no differences in DBP among men in quintiles 2 and 3 vs. quintile 1, but there was a lower DBP among men in blood mercury quintiles 4–5 than in the lower quintiles. Among women we found that DBP in quintile groups 2–5 was no lower than DBP in quintile 1 (Wald, p = 0.19). Our results therefore partly support the hypothesis that the relationship between mercury and BP is non-linear.

The lower DBP among men in the upper quintiles of mercury might be explained by several factors; these might be, for example, a more relaxed lifestyle (approximately 55% of all men in the 4th and 5th quintile of mercury lived in villages) or a higher level of physical activity (1/3 of all men below 63 years in the 4th and 5th quintiles are hunters/fishermen) or due to residual confounding from diet.

Our results concerning a negative relation between blood mercury and DBP disagree with studies in populations that have a lower blood mercury concentration [11]. Valera et al. found a near statistically significant positive relation between blood mercury concentration and DBP among Inuit in Nunavik [11]. Unlike Valera, we omitted information on insulin resistance, cholesterol and triglycerides in the multivariate analyses since we found no empirical evidence suggesting that these are confounding factors. However, like Valera et al., we also included information on selenium and PUFA, which is highly recommended [12] since these factors may affect BP in the opposite direction of mercury.

Additional analyses involving n-3 fatty acids instead of the n-3/n-6 fatty acids ratio made no substantial change to the results. However, among men the relations between blood mercury and SBP, and blood mercury and DBP, were statistically significant when n-3 fatty acids (rather than the n-3/n-6 fatty acids ratio) were included, although the estimates were almost similar. The decrease in DBP was still present when including the n-3 fatty acids instead of the n-3/n-6 fatty acids ratio (Beta = −0,04, S.E. = 0.01, p = 0.0007 vs. Beta = −0.04, S.E. = 0.01, p = 0.01), and the SBP showed a statistically significant decrease (Beta = −0.02, S.E. = 0.01, p = 0.04 vs. Beta = −0.02, S.E. = 0.01, p = 0.06) with a quadrupling of blood mercury. When we omitted the ratio of n-3/n-6 fatty acids and selenium from the analyses in Tables 2 and Table 3 there was no substantial change in the results. For example, among men the DBP still decreased in the multivariate linear regression analysis (Beta = −0.03, S.E. = 0.01, p = 0.0006) as compared to the analyses where we included the n-3/n-6 fatty acids ratio, selenium, and other confounders (Beta = −0.04, S.E. = 0.01, p = 0.001). This supports the findings by Choi [12]; inclusion of PUFA (n-3/n-6 in our study) and selenium influence blood pressure in the opposite direction to mercury. Some may criticise us for not having included the n-3, the n-6 and the ratio of n-3/n-6 fatty acids as confounders in the analyses since these variables all describe different patterns of diet and therefore could have been included in the models from the beginning. We chose not to do so due to the risk of multicollinearity; additional analyses confirmed multicollinearity when including both n-3, the n-6 and the ratio of n-3/n-6 fatty acids (VIF > 10 for n-3 and the ratio of n-3/n-6 fatty acids).

Choi et al. found that methylmercury exposure 7 years previously in 42 Faroese whalers was associated with increased BP. The geometric mean concentration among these whalers was, however, higher than among the male participants in our study (29.5 μg/L vs. 20.5 μg/L). As opposed to Choi et al., we did not include information on PCBs; however PCBs showed no associations with the outcome variables in their study, and PCBs only made a slight difference to the effect of mercury.

We found no systematic relation between SBP and mercury concentration; a relation was only found among men in the analysis when mercury was included as a continuous variable and when adjustments were made for age (p = 0.01). With further adjustment the p-value was 0.06. Neither did we find any relation between quintiles of mercury and SBP in the overall analyses (Wald) or in the pairwise comparisons. Valera et al. found that on inclusion of WC instead of Body Mass Index (BMI) their analysis of mercury and SBP gave a stronger relation [11]. Including BMI instead of WC in our analyses did not change the estimates or S.E. at all – neither among men nor among women (e.g. among men the inclusion of BMI instead of WC in the analyses of the relation between mercury and SBP still gave a negative, but statistically insignificant result (Beta = −0.02, S.E. = 0.01, p = 0.05).

PP is suggested to be a better parameter in the estimation of future cardiovascular disease than SBP and DBP [17]. We found no relation between PP and blood mercury included as a continuous variable at all; however, contrary to many other studies, we analysed the relation split by sex, which may explain the differences. We found a lower PP in the third mercury quintile among women as compared to quintile 1, but how this result should be interpreted is unclear, since DBP was no lower in this quintile group than in the reference group.

In our study the risk of hypertension decreased with increasing blood mercury concentrations (included as a continuous variable) among men only. In the analyses including quintiles of mercury this pattern was, however, not found (Wald test, p = 0.22), which may reflect that there is no dose–response relationship between the level of mercury and hypertension. In a study of the long-term effects of methylmercury exposure on hypertension in Minimata, Yorifuji et al. found a weak dose–response relationship since prevalence of hypertension was lower among participants with medium as opposed to low mercury exposure [18]. A recent large American case–control study, which examined 3,400 cases of cardiovascular disease, with a median toenail mercury concentration of 0.23 μg/g [5] (a relatively low concentration compared with the mean blood concentration in our population (17 μg/L)) did not reveal any adverse effects of mercury exposure. However, the study found that there was a relation between a higher mercury concentration (as opposed to lower levels of mercury) and a lower risk of cardiovascular disease. However an adjusted analysis rendered this trend insignificant. The lack of a relation between exposure to mercury from fish consumption and cardiovascular disease does not, however, preclude the possibility of a mercury-related cardiovascular toxicity at higher levels of mercury exposure.

## Conclusion

We found no adverse associations between whole blood mercury and blood pressure in either men or women. On the contrary, our study showed a negative relation between blood mercury concentrations and hypertension and DBP among adult Inuit men. Analyses of quintiles of mercury, and LOESS curves, suggest that the relationship between mercury and BP parameters is non-linear – or may be explained by confounding by exercise or unknown factors. However, owing to the cross-sectional study design we do not know whether having a high whole blood mercury concentration over time would result in higher prevalence of sudden cardiac death or cardiovascular diseases. Therefore, we recommend investigating this further in a follow-up study of participants with a similar whole blood mercury concentration.

## Endnote

^a^ The high rise in BP only when the BP is measured away from the normal home environment – usually in a clinic.

## Abbreviations

CI, Confidence Interval; BMI, Body Mass Index; DBP, Diastolic blood pressure; OR, Odds ratio; PP, Pulse pressure; S.E, Standard error; SBP, Systolic blood pressure; WC, Waist circumference.

## Competing interests

The authors declare that they have no competing interests.

## Authors’ contributions

ABSN and MD carried out the analyses; ABSN drafted the manuscript; PB was responsible for data collection, participated in the conceptual design, and revision of the manuscript. All authors read and approved the final manuscript.
